# New Developments in Anterior Laryngeal Recording Technique During Neuromonitored Thyroid and Parathyroid Surgery

**DOI:** 10.3389/fendo.2021.763170

**Published:** 2021-10-29

**Authors:** Cheng-Hsin Liu, Tzu-Yen Huang, Che-Wei Wu, Jia Joanna Wang, Ling-Feng Wang, Leong-Perng Chan, Gianlorenzo Dionigi, Feng-Yu Chiang, Hsin-Yi Tseng, Yi-Chu Lin

**Affiliations:** ^1^ International Thyroid Surgery Center, Department of Otolaryngology-Head and Neck Surgery, Kaohsiung Medical University Hospital, Faculty of Medicine, College of Medicine, Kaohsiung Medical University, Kaohsiung, Taiwan; ^2^ Department of Otolaryngology-Head and Neck Surgery, Kaohsiung Municipal Siaogang Hospital, Kaohsiung Medical University Hospital, Faculty of Medicine, College of Medicine, Kaohsiung Medical University, Kaohsiung, Taiwan; ^3^ Department of Biological Science and Technology, National Yang Ming Chiao Tung University, Hsinchu, Taiwan; ^4^ Center for Liquid Biopsy and Cohort Research, Faculty of Medicine, College of Medicine, Kaohsiung Medical University, Kaohsiung, Taiwan; ^5^ Department of Otolaryngology-Head and Neck Surgery, Kaohsiung Municipal Tatung Hospital, Kaohsiung Medical University Hospital, Faculty of Medicine, College of Medicine, Kaohsiung Medical University, Kaohsiung, Taiwan; ^6^ Division of Surgery, Istituto Auxologico Italiano IRCCS, Piazzale Brescia, Milan, Italy; ^7^ Department of Pathophysiology and Transplantation, University of Milan, Milan, Italy; ^8^ Department of Otolaryngology-Head and Neck Surgery, E-Da Hospital, Kaohsiung, Taiwan; ^9^ School of Medicine, College of Medicine, I-Shou University, Kaohsiung, Taiwan

**Keywords:** voice, vocal fold paralysis, thyroid surgery, intraoperative neural monitoring (IONM), recurrent laryngeal nerve (RLN), laryngeal electromyography (EMG), anterior laryngeal recording, thyroid cartilage recording

## Abstract

A recurrent laryngeal nerve (RLN) injury resulting in vocal fold paralysis and dysphonia remains a major source of morbidity after thyroid and parathyroid surgeries. Intraoperative neural monitoring (IONM) is increasingly accepted as an adjunct to the standard practice of visual RLN identification. Endotracheal tube (ET) surface recording electrode systems are now widely used for IONM; however, the major limitation of the clinical use of ET-based surface electrodes is the need to maintain constant contact between the electrodes and vocal folds during surgery to obtain a high-quality recording. An ET that is malpositioned during intubation or displaced during surgical manipulation can cause a false decrease or loss of electromyography (EMG) signal. Since it may be difficult to distinguish from an EMG change caused by a true RLN injury, a false loss or decrease in EMG signal may contribute to inappropriate surgical decision making. Therefore, researchers have investigated alternative electrode systems that circumvent common causes of poor accuracy in ET-based neuromonitoring. Recent experimental and clinical studies have confirmed the hypothesis that needle or adhesive surface recording electrodes attached to the thyroid cartilage (transcartilage and percutaneous recording) or attached to the overlying neck skin (transcutaneous recording) can provide functionality similar to that of ET-based electrodes, and these recording methods enable access to the EMG response of the vocalis muscle that originates from the inner surface of the thyroid cartilage. Studies also indicate that, during surgical manipulation of the trachea, transcartilage, percutaneous, and transcutaneous anterior laryngeal (AL) recording electrodes could be more stable than ET-based surface electrodes and could be equally accurate in depicting RLN stress during IONM. These findings show that these AL electrodes have potential applications in future designs of recording electrodes and support the use of IONM as a high-quality quantitative tool in thyroid and parathyroid surgery. This article reviews the major recent developments of newly emerging transcartilage, percutaneous, and transcutaneous AL recording techniques used in IONM and evaluates their contribution to improved voice outcomes in modern thyroid surgery.

## Introduction

### Neural Monitoring for Improving Voice Outcomes After Thyroid Surgery

Although thyroid surgery is among the most common and safe interventions in endocrine surgery, the risk of complications is still evitable due to the unique anatomical structure and physiological function of the thyroid gland (1). Recurrent laryngeal nerve (RLN) paralysis remains a common thyroid surgery complication that can cause dysphonia, aspiration, and, in some cases, interference with breathing. Therefore, RLN paralysis is a common cause of litigation after thyroid surgery in the current era in which quality of life change is included in assessment of surgical outcome (2, 3). One major change in the past two decades is the growing acceptance of intraoperative neural monitoring (IONM) as a tool for minimizing surgical risk by assisting surgeons in the early localization and identification of the RLN and as a tool for assisting clinical decision-making by enabling real-time monitoring of evoked electrophysiologic laryngeal electromyography (EMG) responses during thyroid surgery.

### Advantages and Disadvantages of EMG Endotracheal Tube Recording for IONM

Currently, EMG recording during neuromonitored thyroid surgery is almost always performed using endotracheal tube (ET) surface electrodes placed in contact with vocal folds during intubation for general anesthesia because of the advantages including their noninvasiveness, wide commercial availability, and capacity to monitor larger areas of the target muscle (4, 5). However, maintaining consistent and stable contact between the ET-based surface electrodes and vocal folds during surgery has become the major clinical challenge to obtain a high-quality recording. Improper positioning of the ET during intubation is not uncommon and may result from an ET that is undersized, rotated, or inserted to the incorrect depth (6, 7). During surgical manipulation, an ET may be displaced by tracheal and neck extension, which inevitably raises the possibility of rotation or depth change (8–10). Both malpositioning and displacement can cause a false decrease or loss of EMG signal. Since false signals may be difficult to distinguish from EMG signal changes caused by true RLN injuries, they may lead to inappropriate surgical decision making. During IONM with ET recording, a malpositioned or displaced ET requires adjustment by the anesthesiologist, which can be troublesome and time-consuming. Intraoperative adjustment of the ET is particularly disruptive in remote thyroid surgery and is virtually impossible in procedures such as transoral robotic approach (11). Additionally, EMG changes have a larger impact in continuous IONM (C-IONM) compared to conventional intermittent IONM (I-IONM) because unstable or shifting baseline EMG cause a C-IONM system malfunction and limit its use for early identification of RLN lesions (12, 13). In addition to the above issues of malpositioning or displacement that may occur during surgical manipulation, other disadvantages of ET include its high expense, the potential for accumulation of saliva to interfere with signal acquisition, and its limited use in pediatric patients and patients with airway abnormality.

### Alternative Electrode Systems That Circumvent the Factors Affecting ET Recording

**Figure 1:**
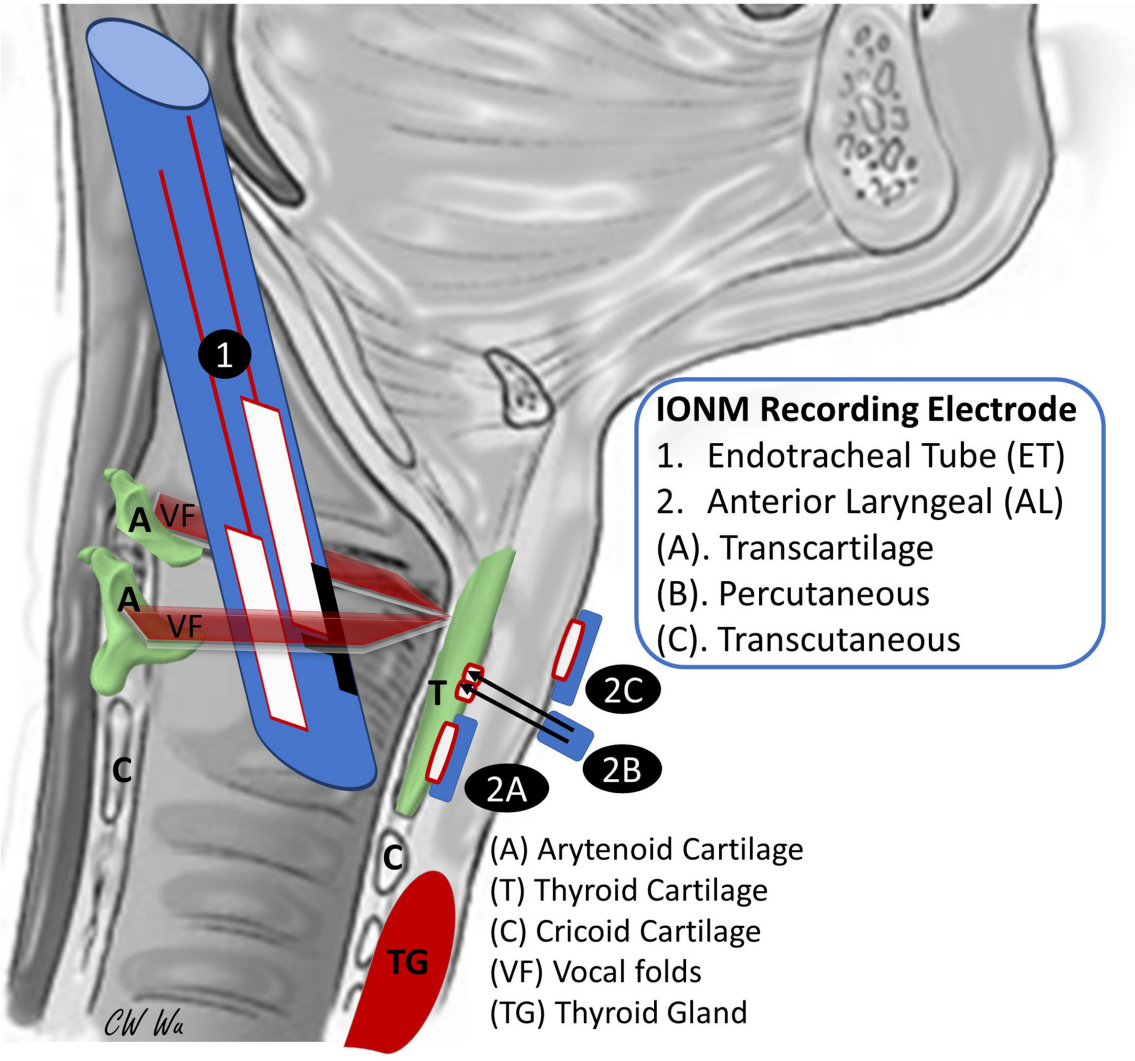
Anatomic relationship between the thyroid cartilage (TC) - vocal folds and intraoperative neural monitoring (IONM) recording electrode placement in different electromyography (EMG) recording techniques used in thyroid and parathyroid surgery. The vocal fold (VF, vocalis and thyroarytenoid muscle) originates from the inner surface of the TC and inserts to the arytenoid cartilage (A). Conventional endotracheal tube (ET) surface electrodes are designed to be placed in contact with vocal folds during intubation for general anesthesia. However, potential displacement of the ET electrode during surgery may affect signal quality and stability. Recent studies have confirmed that anterior laryngeal (AL) transcartilage, percutaneous, and transcutaneous surface recording electrodes placed on the TC outer surface also enable access to the EMG response of the vocal folds and can circumvent factors that negatively affect ET electrode performance.

To avoid the time-consuming processes of verifying and readjusting the ET position during neuromonitored thyroid surgery and to minimize the safety hazard of signal inconsistency and instability. Regarding the basis of anatomy, there are many innovative hypotheses and possible solutions including novel electrodes designs have been proposed. For example, many experimental (14–18) and clinical studies (19–28) have confirmed the hypothesis that needle or adhesive surface recording electrodes attached to the thyroid cartilage (transcartilage or percutaneous recordings) or attached to the overlying neck skin (transcutaneous recording) can function like ET electrodes by enabling access to the EMG response of the vocal fold muscles (vocalis muscle and thyroarytenoid muscle) originating from the inner TC surface **(** **Table 1**, **Figure 1** **)**. These studies have also demonstrated that transcutaneous or transcartilage anterior laryngeal (AL) recording electrodes are as accurate as ET-based surface electrodes in depicting RLN stress during IONM. However, transcutaneous or transcartilage AL recording electrodes could be more stable than ET-based surface electrodes during surgical manipulation on the trachea. These findings indicate that AL electrodes have potential applications in future designs of recording electrodes and support the use of IONM as a high-quality quantitative tool in thyroid and parathyroid surgery. This article reviews recent studies of new and emerging transcutaneous or transcartilage AL recording techniques used during IONM and compares these techniques in terms of their contribution to improved voice outcomes after modern thyroid surgery.

**Table 1:** Current published papers on AL EMG recording technique during neuromonitored thyroid and parathyroid surgery.

Technique	Study design	Author, year	Electrode form	Number of subject	Highlights
**Transcartilage**	Experimental	Wu et al. 2018 (14)	Two disposable adhesive pre-gelled ECG surface electrode on bilateral TC	Porcine model(12 pigs and 24 RLNs at risk)	A proof of concept for transcartilage technique. Confirm the stability and accuracy by trachea displacement and traction injury experiments.
		Zhao et al. 2019 (16)	Two disposable paired subdermal needle electrode (12-mm long, uninsulated) on bilateral TC	Porcine model(4 pigs and 8 RLNs at risk)	Test and identify an optimal site for placement of needle electrodes.
		Zhao et al. 2021 (18)	Two disposable adhesive pre-gelled ECG surface electrode on bilateral TC	Porcine model(4 pigs and 8 RLNs at risk)	Determine the optimal placement locations and sizes of adhesive electrodes.
	Clinical	Chiang et al. 2017 (19)	Two disposable single subdermal needle electrode (12-mm long, uninsulated) on bilateral TC	Comparative Study. Open thyroidectomy(110 patients, 205 RLNs at risk)	First clinical study on needle transcartilage approach and report it obtain higher and more stable EMG signals as well as fewer false EMG results as compared to ET recording.
		Liddy et al. 2018 (20)	One disposable paired adhesive laryngeal EMG surface electrode on bilateral TC	Comparative Study. Open thyroidectomy and parathyroidectomy (15 patients, 20 RLNs at risk)	Demonstrate the transcartilage technique is useful and offer significantly more robust monitoring of the EBSLN.
		Van Slycke et al., 2019 (21)	One disposable paired laryngeal EMG surface electrode suture fixed on bilateral TC	Comparative Study. Open thyroidectomy (25 patients, 25 RLNs at risk)	Confirm the transcartilage technique can obtain higher amplitudes after stimulating RLN and also EBSLN.
		Chiang et al., 2020 (22)	Two disposable paired subdermal needle electrode (12-mm long, uninsulated) on bilateral TC	Case series. Open thyroidectomy (100 patients, 200 RLNs at risk)	Report an optimal technique of needle placement by inserting into the TC subperichondrium from the anterior margin of the thyrohyoid muscle with a slope of 10 to 15 degree.
		Jung et al., 2020 (23)	One disposable paired twisted subdermal needle electrode (22-mm, uninsulated) on bilateral TC	Comparative Study. Open thyroidectomy (38 patients, 54 RLNs at risk)	Report the positive predictive values of loss of signal in ET and TC electrodes were 40% and 100%, respectively.
		Lee et al., 2021 (24)	One disposable paired subdermal needle electrode (12-mm long, uninsulated) on ipsilateral TC	Case series. Unilateral hemithyroidectomy (34 patients, 34 RLNs at risk)	Introduce an alternative method with the advantage of minimal exposure of the TC lamina during unilateral hemithyroidectomy.
		Türk et al., 2021 (27)	One disposable paired twisted subdermal needle electrode (22-mm, uninsulated) on bilateral TC	Case-control study. Open thyroidectomy (885 patients, 1717 RLNs at risk)	The first case-control study to compare ET and TC electrodes, and concluded that TC electrodes are an inexpensive and efficient alternative to ET electrodes.
		Huang et al. (28)	Two disposable paired subdermal needle electrode (12-mm long, uninsulated) on bilateral TC	Comparative Study. Open thyroidectomy (33 pediatric patients, 58 RLNs at risk)	First pediatric study. TC electrodes show excellent stability and quality of EMG signals, and can be a preferable monitoring method for pediatric thyroid surgery
**Percutaneous**	Experimental Clinical	Huang et al., 2020 (17)	Two disposable paired subdermal needle electrodes (25 and 38-mm long, insulated to within 5 mm of tip) percutaneously inserted to TC	Porcine model. (4 pigs, 8 RLNs at risk), 1 case of Transoral robotic thyroidectomy	A proof of concept for percutaneous technique in remote endoscopic or robotic thyroidectomy without neck incision wound.
	Clinical	Li et al., 2020 (25)	Two disposable paired subdermal needle electrode (12-mm long, uninsulated) percutaneously inserted to TC	Case series. Minimally invasive unilateral parathyroidectomy (20 patients, 20 RLNs at risk)	Verify this technique is a feasible, convenient, reliable, and inexpensive method for thyroid or parathyroid surgery with small incision wound.
**Transcutaneous**	Experimental	Wu et al., 2018 (15)	Two disposable adhesive pre-gelled ECG surface electrode	Porcine model (12 pigs, 24 RLNs at risk)	A proof of concept for transcutaneous technique. Confirm the stability and accuracy by trachea displacement and traction injury experiments.
	Clinical	Lee et al., 2020 (26)	Two disposable adhesive pre-gelled EMG surface electrode	Comparative Study. Open thyroidectomy (30 patients, 39 RLNs at risk)	The first published clinical study verifies the usefulness of transcutaneous technique using adhesive skin electrodes.
	Experimental Clinical	Shin et al., 2021 (29)	Two disposable adhesive pre-gelled EMG surface electrode	Porcine model (4pigs, 8 RLNs at risk) Comparative study. Open thyroidectomy (78 patients, 115 RLNs at risk)	Adhesive skin electrode was feasible in both animal models and human patients. Adhesive skin electrode was suggested to the lateral side of the thyroid cartilage lamina closer to the cricoarytenoid joint.

AL, anterior laryngeal; EMG, electromyography; ECG, Electrocardiography; TC, thyroid cartilage; ET, endotracheal tube; RLN, recurrent laryngeal nerve.

## Transcartilage Anterior Laryngeal Recording

**Figure 2:**
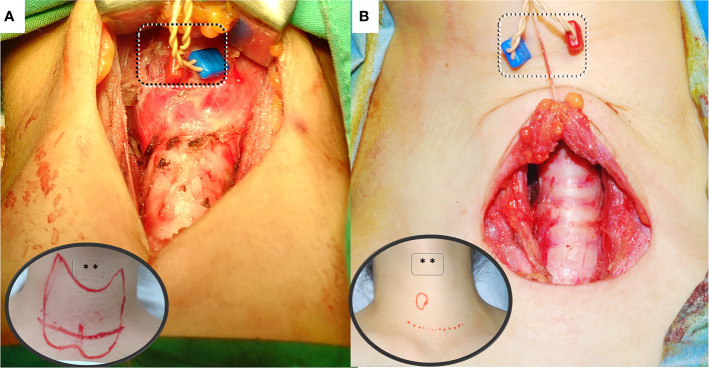
Anterior laryngeal (AL) recording electrode placement for intraoperative neural monitoring. **(A)** Transcartilage Anterior Laryngeal Recording: Skin flaps are elevated superiorly to expose the thyroid cartilage. Needle electrodes are placed onto the subperichondrium layer of thyroid lamina. Inserting the needle at the anterior margin of the thyrohyoid muscle with a 10- to 15-degree angle from the surface of lamina. **(B)** Percutaneous Anterior Laryngeal Recording: The needle electrodes are percutaneously inserted and fixed onto the perichondrium layer of thyroid cartilage. It may be applied in surgeries with lower/smaller incision wound or in remote surgery. However, excessive traction may cause percutaneous needle displacement during the surgery.

The trans-cartilage recording system has yielded many novel surgical applications of IONM technology in recent years. Since vocal fold muscles are innervated only by the RLN and are attached to the anterior inner surface of the thyroid cartilage (TC), surface recording electrodes placed on the outer surface of TC should enable access to EMG signals evoked in vocal fold muscles during IONM (**Figure 1** and **Figure 2A** **) (** 14). To test this hypothesis, Wu et al. (14) performed an animal study using 12 male piglets under standard IONM settings. Adhesive pre-gelled EMG electrodes were attached to the outer surface of the TC for transcartilage AL recording. Evoked EMG signals detected by TC electrodes were compared with those detected by ET electrodes. Typical evoked laryngeal EMG waveforms for the vagus nerve (VN) and RLN were obtained under 1 mA stimulation. Experimental displacements and surgical manipulations of the trachea confirmed the stability and consistency of transcartilage method. Additionally, RLN traction experiments confirmed that transcartilage method accurately reflected neurophysiologic events (14). Zhao et al. (18) investigated the feasibility, EMG stability, and optimal location and size of adhesive surface arrays attached to the TC for use in IONM. Their experiments confirmed that, during surgical manipulations, EMG profiles obtained by transcartilage recording method were more stable than those obtained by ET recording method.

Similarly, Zhao et al. (16) performed an experimental porcine model to test the transcartilage recording by placing needle electrodes on the TC in a porcine model. They confirmed that a perichondral needle electrode can be safely inserted into the avascular area of the TC. Comparing to ET electrodes, TC electrodes registered earlier changes in EMG amplitudes when the nerve is in traction injury. Additionally, TC electrodes obtained higher and more stable EMG amplitudes. Their findings indicate that transcartilage recording electrodes have superior function, easier placement, and lower cost compared to ET electrodes (16). Researchers are increasingly discussing clinical applications of transcartilage AL recording in IONM, and several methods of affixing electrodes to the TC have been reported. For example, one proposed method is to perform transcartilage AL recording using a commercially available 12mm subdermal single needle electrode. Chiang et al. (19) analyzed 205 at-risk RLNs in 110 patients in a clinical comparison of EMG signals recorded by ET and TC surface electrodes in the standardized monitored thyroidectomy. A pair of one-channel electrodes were inserted into the perichondrium of the TC lamina on bilateral side. According to their comparisons, transcartilage AL recording and ET recording had comparable efficacy and reliability during monitored thyroidectomy. In contrast with the complicated and time-consuming procedures for adjusting ET electrodes and verifying their proper position, the setup procedure for transcartilage AL electrodes can be performed quicker (approximately 2 minutes) and easier. Additionally, compared to ET recording, transcartilage AL recording obtains higher and more stable EMG signals during IONM as well as fewer false EMG results. Chiang et al. (22) developed a technique for using two-channel paired subdermal needle electrodes for transcartilage AL recording. Based on their experience in performing the technique in 100 consecutive monitored total thyroidectomies, the authors concluded that, in clinical practice, the technique can be performed without ET electrodes and that it provides high sensitivity and stability of EMG signals. Therefore, it improves the safety and reliability of thyroid surgery and is particularly suitable for use in C-IONM. Another recent study by Jung et al. (23) reported the efficacy of transcartilage recording during standardized monitored thyroidectomy. A pair of 22-mm twisted needle electrodes was attached to the TC in 38 patients with 54 at-risk RLNs. The positive predictive values of loss of signal were 40% and 100% for ET and TC electrodes, respectively. In 2021, Lee et al. (24) reported the advantage of minimizing exposure of one side of the TC by applying a single ipsilateral transcartilage needle electrode during unilateral monitored hemithyroidectomy. Additionally, Türk et al. (27) reported the first case-control study to compare ET and TC electrodes and concluded that TC electrodes are an inexpensive and effective alternative to ET electrodes.

In a prospective clinical cohort study of 25 patients, Van Slycke et al. (21) affixed electrodes directly to the TC perichondrium with two stitches. Compared to ET electrodes, the TC electrodes obtained higher EMG amplitudes induced by stimulation of the VN, RLN and external branch of the superior laryngeal nerve (EBSLN). Liddy et al. (20) proposed and evaluated the use of adhesive dragonfly bipolar surface electrodes (Neurovision Medical, Inomed, Medtronic & Stryker) in 15 consecutive patients undergoing monitored thyroid and parathyroid surgery. Their approach entailed cutting the adhesive electrode in half to create two recording surfaces and then positioning the electrodes over the TC on either side of the midline. The electrodes were then secured by suturing them to the perichondrium. Their experiments again demonstrated that, compared to ET electrodes, adhesive transcartilage AL electrodes provide EMG signals with comparable stability and amplitude and have comparable sensitivity in recording evoked responses during IONM. Additionally, since adhesive transcartilage AL electrodes are accessible in the operative field, they are easily monitored and controlled by the surgeon, and they are unaffected by potential ET displacement during surgery (20). Finally, since transcartilage AL electrodes enable robust EBSLN monitoring, they reduce the risk of high-pitched voice function loss in thyroid and parathyroid surgery (30).

In pediatric patients, anatomical characteristics, e.g., smaller RLN diameter compared to adults, can make IONM challenging. One consensus statement suggested that IONM is beneficial in pediatric patients with a bulky thyroid and lymph node disease (31). Since the larynx and trachea are relatively small in pediatric patients, the limited selection of commercially available ET electrode sizes combined with the increased difficulty of confirming ET electrode position in pediatric patients may result in unstable and inconsistent contact between the ET electrode and the vocal fold (28, 32). Huang et al. (28) compared electrode types in 33 pediatric patients who had received neuromonitored thyroid surgery and concluded that, compared to EMG signals obtained by ET electrodes, signals obtained by TC electrodes had superior amplitude, stability, and quality, which greatly facilitates the meticulous RLN dissection required in pediatric thyroidectomies, especially in pediatric patients with thyroid cancer.

## Percutaneous Anterior Laryngeal Recording

The feasibility of using TC perichondral needle electrodes as recording electrodes and their good signal stability have been well established in many animal and clinical studies. However, these electrodes require a standard neck incision for open exposure of the TC. That is, TC perichondral needle electrodes are inapplicable when invasiveness must be minimized and in remotely-performed endoscopic or robotic thyroidectomy. To overcome this limitation, Huang et al. (17) designed a novel IONM percutaneous recording method for remotely performed thyroid surgery **(** **Figure 1** and **Figure 2B** **)** in which pairs of insulated needle electrodes (lengths, 25 mm and 38 mm) were inserted percutaneously into the TC perichondrium to within 5 mm of the tip. The four most widely used remote thyroidectomy techniques (bilateral axillary-breast, transoral, transaxillary, and retroauricular approaches) were evaluated in animal experiments and in their initial case series. They concluded that percutaneous TC recording technique is feasible and can be modified according to the approach used for remote access. This technique reduces interruption of the surgical procedure while still providing reliable EMG signals (17).

Percutaneous AL recording during IONM in minimally invasive parathyroidectomy was recently reported by Li, et al. (25). The authors used paired 12mm long needle electrodes for percutaneous recording in 20 patients and successfully detected typical EMG signals. Based on their findings, the authors concluded that their technique is feasible, convenient, reliable, and cost-effective when IONM is used to assist minimally invasive thyroid or parathyroid surgery (25).

## Transcutaneous Anterior Laryngeal Recording

In addition to transcartilage AL recording, transcutaneous AL recording is another innovative procedure that is proposed for modern IONM technology (15). **Figure 1** shows that transcartilage recording is effective for evaluating vocal fold muscle function and RLN function in TC, and transcutaneous recording may be effective for the same purpose.

Technical advances in epidermal electronics now enable fabrication of sensors in the ideal form can be affixed as an electronic second skin. In 2018, Wu et al. used a porcine model with well-established applicability in IONM research to verify the hypothesis of transcutaneous AL recording. Electrically evoked EMGs were recorded from surface electrodes attached to the ET and from adhesive pre-gelled surface electrodes (Neotrode II^®^-ConMed) attached to the anterior neck skin. In their experiments, ET electrodes and neck adhesive skin electrodes successfully recorded typical evoked laryngeal EMG waveforms from RLNs and VNs under 1 mA stimulation. Additionally, both electrode types accurately detected adverse EMG events under experimentally induced RLN traction stress. Under experimentally induced tracheal displacement, however, EMG signals obtained by ET electrodes widely varied whereas EMG signals obtained by transcutaneous electrodes were stable (15). Although this proof-of-concept study confirmed the stability and accuracy of EMG signals obtained by transcutaneous approach, it also revealed the need for new electrode designs that provide more consistent and accurate EMG amplitudes before practical clinical application of this approach.

In 2020, Lee et al. (26) performed the first clinical study to evaluate the efficacy of transcutaneous AL recording during monitored thyroidectomy. A disposable pre-gelled adhesive surface electrode (1.5 cm x 2.0 cm x 2.5 cm; DSE3125; Medtronic Xomed; Jacksonville, FL, USA) was attached to each of the two upper margins of the TC surface. The setup time for the skin electrodes was less than 1 minute in all cases. Their experimental results confirmed the effectiveness of IONM for recording evoked biphasic EMG signals of acceptable quality in all at-risk nerves.

Recently, Shin et al. (29) investigated the optimal attachment location of transcutaneous adhesive skin electrodes for IONM. In porcine animal model, the mean amplitude obtained using laterally attached transcutaneous electrodes was significantly higher than that obtained using medially attached skin surface electrodes. However, there was no significant difference in amplitude according to vertical levels (upper/middle/lower). In human patients, they reported that the ET electrode (716.25 ± 543.35 µV) showed a significantly higher mean amplitude than laterally attached transcutaneous electrodes (258.48 ± 77.31 µV), and the laterally attached transcutaneous electrodes showed a significantly higher mean amplitude than the medially attached skin electrodes (185.22 ± 66.56 µV) on RLN stimulation. Therefore, they concluded that transcutaneous recording is feasible and the lateral side of the thyroid cartilage lamina may be better than the medial side for electrode attachment to obtain better EMG signals from the intrinsic laryngeal muscles.

## Discussion

Although thyroid surgery is now a commonly performed procedure worldwide, RLN injuries are still common complications and a major cause of low satisfaction with thyroid surgery outcomes and medical malpractice litigation. Use of IONM in thyroid surgery has become well established in the past two decades, and surgical applications of IONM are increasingly accepted throughout the world. Recent registry-based studies performed in Sweden (SQRTPA) (33), Europe (EUROCRINE^®^) (34), United Kingdom (UKRETS) (35), and other regions (36) provide insight into current IONM practices. Most studies agree that a large and growing majority of thyroid surgeons currently use IONM for anatomical identification of the RLN and for evaluating RLN injury. Another common motivation for using IONM is to reduce the risk of temporary and permanent RLN paralysis.

The most important issue when IONM is performed using an ETT as the recording side is that IONM has a high negative predictive value (92%-100%) but a relatively low and variable positive predictive value (10%-90%) (37). The IONM recording quality (i.e., stability and consistency) depends on the stability of contact between the ET electrodes and the vocal folds, and contact quality may vary widely during surgical manipulations. Therefore, alternative electrode types such as transcartilage, percutaneous, and transcutaneous AL recording electrodes have been extensively studied in recent years to minimize factors that negatively affect ET-IONM accuracy and efficacy. This article presents a complete overview of state-of-the-art transcartilage, percutaneous, and transcutaneous AL recording techniques used in IONM and compares their contribution to improved voice outcomes in modern thyroid surgery.

**Figure 3:**
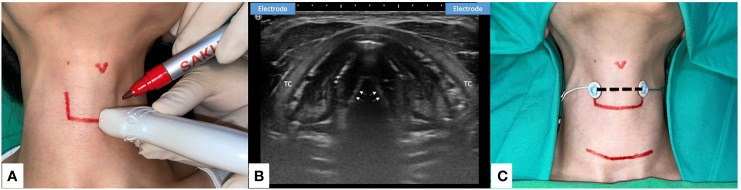
Transcutaneous Anterior Laryngeal Recording electrode placement for Intraoperative neural monitoring. **(A)** Preoperative skin marking: lateral border of thyroid cartilage and the level of true vocal fold. Precise localization may be done with the application of ultrasound (US). **(B)** The axial view of true vocal folds (white arrow). Lateral side of the thyroid cartilage (TC) lamina is the optimal location to place the skin surface electrodes (US illustration). **(C)** Transcutaneous recording electrodes are placed at the level of true vocal fold (dotted line). Transcutaneous recording method is applicable in small incision wound or remote thyroid surgery. Skin flap beneath the surface electrodes should be avoided.

This is the first comprehensive review in the literature of IONM with transcartilage (14, 16, 18–24, 27, 28), percutaneous (17, 25), and transcutaneous (15, 26) AL recording electrodes. The techniques are illustrated (**Figure 1**), major findings of each study are summarized and highlighted (**Table 1**), and the pros and cons of each method are discussed and compared (**Figure 2**, **Figure 3** and **Table 2**). In comparison with conventional ET recording IONM, advantages of AL recording IONM reported in both animal and clinical studies include lower invasiveness, better quality and stability of EMG signals, and avoidance of time-consuming disturbances such as the need to verify the ET position. In addition, the high reliability and quality of EMG response making these recording methods not only useful during routine ordinary thyroid and parathyroid surgery, but also practical during thyroid surgery with small incision, remote access, and in specific (e.g., pediatric) populations. In addition to ensuring stable EMG signals, AL recording IONM has several advantages. First, the equipment setup is user-friendly; reported setup times approximate 2 minutes for transcartilage needle electrodes (19) and less than 1 minute for transcutaneous electrodes (26). Second, only a short setup time is required for rapid implementation when an unexpected need for IONM occurs during surgery. Third, dislodged electrodes can be identified and managed intraoperatively. Finally, AL electrodes are apparently more cost effective than ET electrodes (28). A pair of adhesive pre-gelled electrodes or needle electrodes can be purchased for the equivalent of less than 100 USD, which is at least five-fold lower than the cost of EMG ET electrodes.

**Table 2:** The pros and cons of different AL EMG recording techniques during neuromonitored thyroid and parathyroid surgery.

Technique	Advantages	Disadvantages
**Transcartilage**	(1). EMG signal shows less affected by surgical manipulation and is comparable sensitive in reflecting a neurophysiologic event* (2). Cost-effectiveness and surgeon-friendly electrode setup* (3). Higher (needle electrode) or comparable (adhesive electrode) EMG amplitudes* (4). Enable of more robust EBSLN monitoring* (5). Suitable for pediatric and tracheostomy cases	(1). Requires an adequate skin flap elevation to expose the thyroid cartilage, limit of use in procedures with small or no neck incision wound. (2). Electrode (needle) insertion may be complicated by scar tissue caused by revision surgery or, in older adults, by calcified TC (3). Intralaryngeal penetration of (needle) electrode may cause laryngeal hematoma, laceration, infection, or rupture of an endotracheal cuff
**Percutaneous**	(1). and (2). same as Transcartilage method (3). High (needle electrode) EMG amplitudes recorded* (4). Feasible and useful for small incision and remote access procedures	(1). Longer electrode (needle) may be required according to different approach of remote access. (2). Electrode (needle) insertion may cause inadvertent injury to the cricothyroid or strap muscle, and can inadvertently record far field potentials. (3). Excessive skin retraction could cause percutaneous electrode displacement.
**Transcutaneous**	(1). and (2) same as Transcartilage/Percutaneous method (3). Biphasic EMG signals of acceptable quality recorded (adhesive skin electrode)	(1) The recorded EMG amplitude of skin electrode is relatively lower* (2) The electrode sensitivity could be affected by patient characteristics (short or obese neck), tumor size, and degree of subplatysmal flap elevation.

*as compared to EMG endotracheal tube recording.

AL, anterior laryngeal; EMG, electromyography; TC, thyroid cartilage; EBSLN, external branch of superior laryngeal nerve.

Whereas these novel techniques are apparently practicable and have no major disadvantages in most clinical applications, disadvantages of transcartilage electrodes include the higher skin flap elevation required to expose the TC (**Figure 2A**), and the difficulty of manipulating the electrodes in procedures performed within a limited operative space, e.g., in endoscopic thyroidectomy or in procedures in which the size of the incision is minimized to improve cosmetic outcome. Additionally, a needle electrode insertion may be complicated by scar tissue caused by revision surgery or, in older adults, by calcified TC, both of which can substantially impair recording of compound muscle action potential responses (14). Finally, patients undergoing such procedures have a low but still risk of laryngeal hematoma, laceration, infection, or rupture of an endotracheal cuff, especially in procedures performed with needle electrodes. Therefore, the recommendation is to insert the needle gently into the subperichondrium of the middle thyroid lamina from the anterior margin of the thyrohyoid muscle with a 10- to 15-degree slope on each side (22).

Although percutaneous AL electrodes are applicable in small incision thyroid and parathyroid surgery and in remote thyroidectomy, insertion of needle electrodes into the TC should be performed with extreme caution to avoid inadvertent injury to the cricothyroid muscle or EBSLN, which can cause muscular hematoma or scar tissue formation and subsequent alterations in vocal pitch. Additionally, percutaneous needle recording electrodes can inadvertently record far field potentials. Therefore, the recommended practices are confirming the stimulation site and comparing corresponding VN, RLN, or EBSLN waveforms, and adjusting the current intensity to minimize recording of far field potentials. Finally, excessive skin retraction or the use of skin retraction for counter-traction during remote thyroidectomy (17) could cause percutaneous needle displacement (**Figure 2B**).

Although transcutaneous AL recording in IONM has proven feasible and reliable, further technical refinement is needed to address some technical flaws. The skin electrode could be affected by patient characteristics such as obese or short neck, degree of subplatysmal flap elevation, and large tumor size. During surgery, subplatysmal flap and retraction of the strap muscle during dissection of the thyroid upper pole may hinder neural signal transmission. The EMG signals actually recorded after upper pole dissection might also be diminished (38). Therefore, this technique may not be useful in procedures that require a large incision or involve a large tumor size (15). Additionally, since both animal and clinical studies agree that low amplitude remains the major limitation of this technique, further research is needed to improve electrode designs and recording quality. Shin et al. (29) suggest that the lower amplitude could be overcome by attaching the skin electrodes more laterally and close to the cricoarytenoid joint. Therefore, the authors of this review suggest the surgeons may consider using ultrasonography to decide the optimal location of the AL electrodes preoperatively (**Figure 3**).

In summary, this review of the recent literature regarding the feasibility, stability, safety, and efficiency of new and emerging techniques for obtaining transcartilage, percutaneous, or transcutaneous AL recordings during IONM found that recently developed techniques increase the potential applications of IONM as a high-quality quantitative tool in thyroid and parathyroid surgery. However, this review also revealed that most clinical studies have enrolled a relatively small number of patients. Therefore, future prospective studies in larger populations are needed to explore and verify the applicability and benefits of these techniques. Several novel techniques that can further improve AL recordings have been recently reported in the literature. One study uses a nanosheet-based microneedle for EMG recording, which minimizes the risk of laryngeal injury caused by needle insertion (39). Instead of using electrophysiologic laryngeal EMG to evaluate muscle movement, several recent animal studies have also evaluated the use of novel devices that use a piezo-electric surface pressure sensor (40, 41) or an accelerometer sensor (42) to measure muscle twitch. Another interesting development is an ‘‘electronic skin’’ (e-skin) fabricated by embedding various serpentine sensors in a highly stretchable net sandwiched between two protective layers of equal thickness (43). Like a bandage, the device can be attached to the skin surface to acquire physiological information that may be applicable in transcutaneous AL recording. In the future, these sensors may be integrated in various transcutaneous, transcartilage or ET-base surface electrode designs for IONM recording during thyroid surgery.

## Conclusion

In the current era in which quality of life is increasingly included as a surgical outcome measure, voice outcome after thyroidectomy is an important consideration for both patients and any clinician involved in managing such patients. Several methods and technologies are being developed to overcome the limitations and disadvantages of the current IONM system that relies on EMG ET recording. This article reviews major recent developments and progress in new and emerging transcartilage, percutaneous, and transcutaneous AL recording techniques used during IONM. In comparison with the conventional ET recording method, many experimental and clinical studies have shown that these up-to-date AL recording methods have advantages of lower invasiveness, better EMG quality and stability, and avoidance of time-consuming disturbances for ET position verification. In addition, these methods are not only feasible for routine ordinary thyroid and parathyroid surgery, but also practical during thyroid or parathyroid surgery with a small incision, remote access, and in pediatric operations. Additional advantages of AL recordings include a cost-effective and surgeon-friendly set-up, allow for rapid implementation when an unexpected need, and the dislodged electrodes can be easily identified and managed intraoperatively. Although there are still some limitations of each recording method, we believe the continuous practical application and implementation of these developments will further optimize IONM to the ultimate benefit of thyroid surgery patients.

## Author Contributions

CL, TY-H, CW-W, and GD conceived and designed the study. Administrative support was obtained by LF-W, HY-T, and YC-L. Provision of study materials by JW, L-PC, H-YT, Y-CL had collected and assembled the data. Data analysis and interpretation was done by F-YC, T-YH, and CL. All authors contributed to the article and approved the submitted version.

## Funding

This study was supported by grants from Kaohsiung Medical University Hospital (KMUH109-9M44), Kaohsiung Municipal Siaogang Hospital/Kaohsiung Medical University Research Center grants (KMHK-DK (C)110009, I-109-04, H-109-05, I-108-02) and Ministry of Science and Technology (MOST 108-2628-B-037-006, MOST 109-2628-B-037-014, MOST 110-2314-B-037-104-MY2, MOST 110-2314-B-037-120), Taiwan.

## Conflict of Interest

The authors declare that the research was conducted in the absence of any commercial or financial relationships that could be construed as a potential conflict of interest.

## Publisher’s Note

All claims expressed in this article are solely those of the authors and do not necessarily represent those of their affiliated organizations, or those of the publisher, the editors and the reviewers. Any product that may be evaluated in this article, or claim that may be made by its manufacturer, is not guaranteed or endorsed by the publisher.
